# Autoimmune Inner Ear Disease: The Current State of Knowledge

**DOI:** 10.3390/audiolres16050159

**Published:** 2026-10-18

**Authors:** Karol Myszel, Piotr Henryk Skarżyński, Tomasz Michalak

**Affiliations:** 1 Center of Hearing and Speech, 7 Mokra Street, 05-830 Kajetany, Poland; 2 Faculty of Health Sciences, University of Applied Sciences, 2 Popieluszko Street, 62-510 Konin, Poland; 3 Department of Teleaudiology and Screening, World Hearing Center, Institute of Physiology and Pathology of Hearing, 10 Mochnackiego Street, 02-042 Warsaw, Poland; 4 Institute of Sensory Organs, 1 Mokra Street, Kajetany, 05-830 Nadarzyn, Poland; 5 Heart Failure and Cardiac Rehabilitation Department, Faculty of Dental Medicine, Medical University of Warsaw, 8 Kondratowicza Street, 03-242 Warsaw, Poland; 6 Institute of Physiology and Pathology of Hearing, 10 Mochnackiego Street, 02-042 Warsaw, Poland; t.michalak@ifps.org.pl

**Keywords:** autoimmune inner ear disease, sensorineural hearing loss, immunosuppression

## Abstract

**Background/Objectives**: An increasing number of cases of sensorineural hearing loss are caused by autoimmune inflammatory reactions within the cochlea, which can either be limited to the inner ear or associated with other systemic autoimmune reactions. The objective of this work was to conduct a narrative review of the worldwide literature from the past five years, to summarize the current state of knowledge on autoimmune hearing loss, and to understand its pathology and its treatment strategies. As a narrative review, our publication aimed to describe the most interesting and up-to-date case reports that add to our knowledge of autoimmune inner ear conditions. **Methods**: We searched the Scopus, Web of Science, and PubMed databases using the key phrases “autoimmune inner ear disease”, “autoimmune sensorineural hearing loss”, and “sudden sensorineural hearing loss”. The selection covered papers published within the past five years. **Results**: Based on the selection criteria, we identified 122 relevant articles. Further analysis and limitation to the main scope of the review led to the final selection of 48 papers, which formed the basis for this review. **Conclusions**: Autoimmune hearing loss appears to be an increasing clinical problem. The underlying pathology can be limited to the ear; however, it may also occur during the course of other autoimmune processes with multisymptomatic manifestations in different systems and organs. The underlying pathology is an immune-mediated inflammatory response to antigens, including self-antigens located in a patient’s body tissues. This pathology leads to various dysfunctions. Diagnosis is challenging, as there are no specific diagnostic criteria. Inflammatory markers are usually elevated; however, they are not specific enough to determine a final diagnosis. Treatment strategies differ, depending on the symptoms; however, immunosuppressive drugs, mainly corticosteroids (systemic or trans tympanic), appear to be a treatment of choice.

## 1. Introduction

Autoimmune inner ear disease (AIED) is a condition with progressive sensorineural hearing loss, often accompanied by tinnitus, and dizziness or vertigo. It is a result of a dysfunction in the immune system that leads to damage within the inner ear and/or vestibulocochlear nerve, causing fast progressive (weeks to months), usually bilateral, sensorineural hearing loss. Primary AIED includes only the inner ear, whilst secondary AIED is associated with other autoimmune diseases. Hearing loss occurs in around 30% of cases of systemic autoimmune diseases [1,2,3].

The exact pathology of AIED remains unclear. Recent progress in immunology has helped us to understand the underlying mechanisms; however, many studies are still in progress.

Determining a final diagnosis of AIED is still very challenging, as there are no specific markers and no standardized criteria exist; therefore, clinicians should focus on the lab tests, symptoms, detailed medical history, and other available data to obtain a more comprehensive patient profile. Imaging examinations, including MRI, are not deterministic for AIED, although some findings may be helpful in differential diagnosis. AIED symptoms should be differentiated from other clinical conditions, such as sudden hearing loss (usually appearing within less than 72 h), Meniere’s disease, syphilis of the inner ear, meningitis, IgG4 disease, paraneoplastic syndromes, genetic progressive SNHL, lymphoma, and brain metastases involving dura mater. Immunosuppressive treatment, when implemented appropriately, offers a chance for a significant improvement in hearing in AIED patients. Corticosteroids (systemic or trans-tympanal) remain a first-line treatment; however, they are often combined with other drugs for better results. Other treatment schemes are undergoing testing with some good prospects for the future [4].

## 2. Materials and Methods

We searched the Scopus, Web of Science, and PubMed databases using the key phrases “autoimmune inner ear disease”, “autoimmune sensorineural hearing loss”, and “sudden sensorineural hearing loss”. The selection covered the papers published within the past five years. Based on the selection criteria, we identified 122 relevant articles. Further analysis and limitation to the main scope of the review led to the final selection of 48 papers, which form the basis for this review.

**Figure 1:**
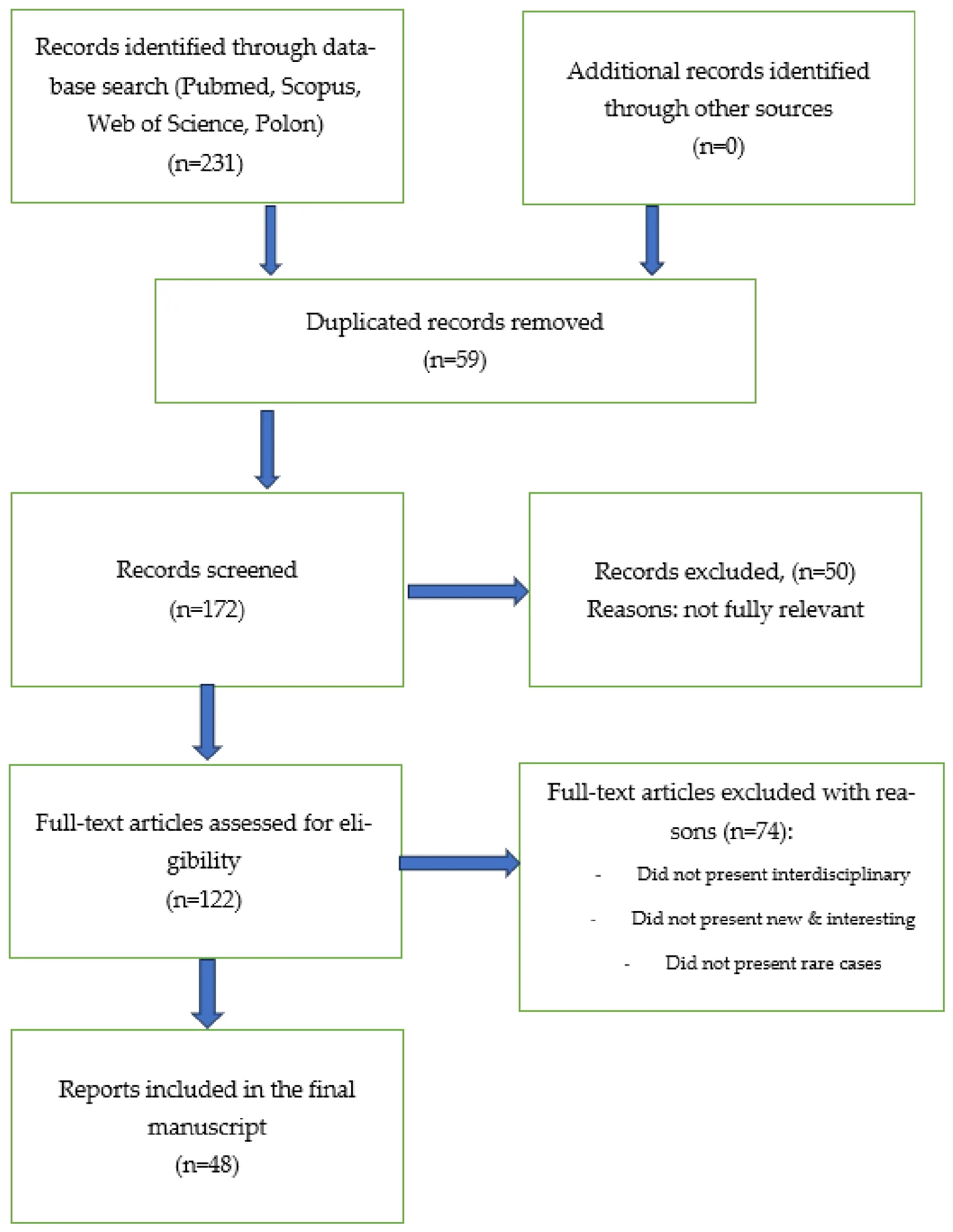
Selection process of articles used in this manuscript.

The selected publications covered 1065 patients, with 634 females and 431 males, at an average age of 51. All selected studies were retrospective. Some publications included a substantial number of individuals, while others were case reports of a limited number or even one patient, which is a limitation of this review. However, due to the variety of possible presentations of AIED, single reports can shed additional light on the discussion; therefore, they were included. The reports came from various medical and research centers worldwide, which adds to the value of this review. The selected studies had very diverse inclusion criteria; however, no specific biases were found. The final selection of papers for this review was based on the following: (a) an overlap with various medical specialties, (b) how the cases offered some new perspectives or filled information gaps, and (c) reports of very rare cases. The selection process is presented in Figure 1.

The review covered papers published within the past five years to ensure the most up-to-date knowledge. On the one hand, the most recent articles refer to older references, and on the other hand, they reflect the newest advances in the immunology of the inner ear.

## 3. Results

Sensorineural hearing loss of autoimmune origin may affect one ear, or it may occur bilaterally. It may be an isolated pathology of the inner ear or be a part of multisymptomatic syndromes. Sreenivas et al. [5] described six patients in whom an autoimmune background for bilateral progressive hearing loss was found; three of them had a primary ear disease with only ear symptoms, whilst the remaining three had hearing loss over the course of systemic autoimmune conditions with audio-vestibular symptoms. Two of them developed facial paralysis, and two other patients had systemic features (one case of Wegener’s granulomatosis and one case of relapsing ear polychondritis with the redness of pinna). All patients presented an increased erythrocyte sedimentation rate (ESR) and antinuclear antibodies (ANA). Bustos-Merlo et al. [6] examined 55 patients with AIED. Of these, 87.5% had bilateral sensorineural hearing loss; in 81.25%, this was accompanied by dizziness or vertigo, and in 56.25%, by tinnitus. Most of the patients had increased antiphospholipid antibody levels. Almost 82% of patients were positive for lupus anticoagulant antibodies (LA). In 37% of patients, anticardiolipin antibodies (aCL) were detected, and 32% had anti-beta2 glycoprotein (anti-B2GP) antibodies. Over half of the subjects had positive antinuclear antibodies (ANA). Xu et al. [7] measured the levels of the CD163 protein in patients with sudden sensorineural hearing loss. This protein is a transmembrane expressed on monocytes and macrophages, serving as a receptor for hemoglobin–haptoglobin complexes. The connection between the receptor and the complexes is a tissue-protective mechanism against oxidative damage. Therefore, increased levels of CD163 suggest macrophage activation, which is seen in AIED. Corticosteroid treatment in this study’s subjects reduced the CD163 levels, accompanied by lowering hearing thresholds.

In many cases described in the literature, AIED occurs as one of the symptoms in other autoimmune diseases. Zuccardi et al. [8] described a pediatric case of linear morphea in the left hemisphere of the skull. The patient presented with a hearing threshold increase: a wave V at 55 dB NHL in the right ear and a wave V at 60 dB NHL in the left ear, along with facial asymmetry. The patient was also diagnosed with a lesion in the left parieto-occipital region and an area of hyperpigmentation in the frontoparietal region. The authors concluded there was a possible relationship between systemic or localized autoimmune disease and AIED. Zarachi et al. [9] described autoimmune hepatitis (AΙH) in a patient who parallelly developed rapidly progressive, bilateral, asymmetrical sensorineural hearing loss.

Sjögren’s syndrome is an autoimmune condition characterized by the systemic dysfunction of exocrine glands, mainly salivary and lachrymal. It may occur as an isolated condition (primary) or may be accompanied by systemic lupus erythematosus (secondary). Sensorineural hearing loss is sometimes observed in primary and secondary Sjögren’s syndrome. Okawa et al. [10] described cases of AIED occurring together with Sjögren’s syndrome, confirming the strong coexistence of the two conditions with the autoimmune process as a background. Karunaratne et al. [11] described celiac disease cases accompanied by sensorineural hearing loss. Here, the hearing loss is usually progressive and permanent and requires frequent audiological checkups. Sensorineural hearing loss has often been described in systemic lupus erythematosus (SLE). Similarly, there are cases of sensorineural hearing impairment during Takayasu’s arteritis (TAK); however, more cases need to be examined to better document the relationship between the two conditions, as described by Kimyon et al. [12]. Sun et al. [13] conducted a study aimed at evaluating the association between autoimmune thyroiditis and sudden sensorineural hearing loss in 105 patients. Twenty-six (24.8%) of the hearing-impaired patients had elevated thyroid autoantibodies; hearing improvement was significantly better among patients with lower thyroid autoantibodies. The researchers concluded that thyroid autoimmunity may be a pathogenesis factor for sudden sensorineural hearing loss and associated with more severe hearing loss of high-frequency hearing.

Hearing impairment has been described as one of the symptoms in Susac syndrome. This is a rare, autoimmune, occlusive endothelium pathology that involves blood vessels in the central nervous system, retina, and inner ear, ultimately leading to encephalopathy, cognitive disorders, amblyopia, and sensorineural hearing impairment. The complete set of symptoms is present in less than one-third of patients.

Hung at al. [14] analyzed inner ear symptoms among patients with and without polymyositis and dermatomyositis PM/DM. The study included 1622 patients diagnosed with PM/DM and 8109 patients without PM/DM and showed that patients with PM/DM had higher prevalence rates of sensorineural hearing loss, tinnitus, and vertigo than patients in the control group. Orłowska et al. [15] described Vogt–Koyanagi–Harada syndrome (VKH), which is a rare granulomatous autoimmune disease. The autoimmune response is directed against melanocytes. Antibodies target tissues rich in melanocytes, i.e., skin, meninges, eye and inner ear. In advanced cases with severe inflammation present, permanent visual impairment together with sensorineural hearing loss with tinnitus and vertigo may occur. Other symptoms include nausea, vomiting, neck stiffness, headaches, and vitiligo on the skin of the face, hands, shoulders, and lower back. In some cases, alopecia is present. Wong et al. [16] published a case report of a woman with sudden single-sided sensorineural hearing loss, uveitis, erythematous lower limb lesions, and bilateral pitting oedema. High-resolution computer tomography of the chest, followed by bronchial biopsy, confirmed sarcoidosis. Treatment with oral steroids led to improvements regarding hearing and respiratory symptoms.

Wu et al. [17] described a group of other autoimmune conditions with inner ear manifestation. Multiple sclerosis (MS) is a chronic disease of the central nervous system characterized by inflammatory demyelination and neurodegeneration, which may lead to hearing loss and vestibulopathy. Autoimmune thyroid diseases (AITDs) result from an immunological response against thyroid antigens, mainly thyroid peroxidase (TPO), thyroglobulin (Tg), and the TSH receptor (TRAb), leading to elevated levels of the corresponding autoantibodies (TgAbs, TPOAbs, TRAbs). The inflammation also leads to extra-thyroid symptoms such as hearing loss and vertigo. Behçet’s disease (BD) is a recurrent inflammatory vasculitis that primarily involves the skin, mucous membranes, eyes, joints, nervous system, and blood vessels. Hearing and vestibular dysfunction occurs due to involvement of the auditory and central vestibular pathways. Psoriasis is a chronic immune-mediated systemic inflammatory disorder. Apart from the skin symptoms, it may lead to auditory and vestibular dysfunction. A rare systemic vasculitis named Cogan’s syndrome may also lead to vestibulo-auditory symptoms, as described by Belfeki et al. [18]. An immune-mediated vasculitis targets vessels with autoantibodies, targeting corneal, inner ear, and endothelial antigens. Other symptoms include eye pain, visual impairment, muscle pain, and fever. Busch et al. [19] published an example of autoimmune vasculitis in necrotizing vasculitis with polyangiitis (GPA, formerly referred to as Wegener granulomatosis). The inflammation involves small-to-medium vessels and leads to necrotizing granulomatous inflammation. Advanced cases present with microvascular-mediated glomerulonephritis. Ear symptoms may include otitis media, sensorineural hearing loss, and vertigo. Giant cell arteritis (GCA) is a chronic systemic granulomatous vasculitis. Clinical manifestations usually include headache, jaw claudication, and systemic inflammatory symptoms. GCA is often associated with polymyalgia rheumatica (PMR). Vertigo and other signs of audio-vestibular symptoms are often present, as described by Fontcuberta et al. [20].

Sensorineural hearing loss has also been described in other autoimmune conditions. Ger et al. [21] reported SNHL in psoriatic arthritis, Barradas-Hernández et al. [22] reported it in ankylosing spondylitis, and Saavedra Torres [23] reported the appearance of hearing impairment associated with hypersensitivity reaction type III in serum disease. Bodh et al. and Birda et al. reported cases of sensorineural hearing loss in ulcerative colitis [24,25] and Karadag et al. [26] in nodular arteritis.

The autoimmune diseases described above, with the spectrum of possible manifestations including hearing loss, are presented in Table 1.

## 4. Discussion

### 4.1. Immunology of the Inner Ear

The human inner ear has a complicated and highly specialized anatomical structure. Control of hearing and balance is a complex phenomenon that can be disrupted by various factors, such as viruses, bacteria, noise, older age processes, mechanics, etc. The immune system of the cochlea helps to protect it from damage.

Groups of immune cells are found in the lateral wall and other parts of the cochlea that regulate homeostasis and generate an immunological response, when necessary. Macrophages comprise the most numerous populations of immune cells in the cochlea (above 80% of all cells). They are mainly located in the stria vascularis, spiral ligament, Rosenthal’s canal, and in the spaces among the groups of neurons in the spiral ganglion. Their role in the immune response is mainly antigen presentation. They are also involved in eliminating metabolic debris from within the cells. A strong activation of macrophages takes place when generating an immune response to factors such as pathogens, deoxidation, noise and mechanical damage, intoxication, or older age. In such situations, macrophages are involved in the inflammatory process and in promoting fibrosis. T lymphocytes comprise another population of cochlea immune cells. These cells are recruited and proliferate at the site of inflammation. T regulators are a cell subpopulation responsible for modulating an inflammatory process, whilst T helpers are usually involved in tissue repair and resolution of the inflammatory process [27].

Dendritic cells, part of the group of antigen-presenting cells, also reside in the stria vascularis of the cochlea. They present antigens to T helpers and bind them to the protein CD4, which initiates an immune response. Minor populations of immune cells in the cochlea are represented by B lymphocytes, granulocytes and natural killer cells that are considered to assist in the immune response due to their specialized functions.

The communication process between cells for signaling, cell recruiting, and the modulation of the inflammatory process occurs through the substances produced by the immune cells themselves. Small proteins called cytokines act as pro-inflammatory agents. The largest role is played by interleukin type 6 (IL-6), interleukin type 1β (IL-1β), interleukin type 17 (IL-17), and tumor necrosis factor α (TNF-α). The levels of those proteins are increased in various inner ear pathologies, confirming their importance in the process of inflammation. Inflammation resolving and repair processes are regulated by anti-inflammatory cytokines, such as interleukin type 10 (IL-10), type 22 (IL-22), type 33 (IL-33), and transforming growth factor beta (TGF-β). These cytokines play a crucial role in sustaining the appropriate function of cochlear hair cells by resolving an inflammatory process and ensuring a return to cell homeostasis. The cochlear steady state is therefore achieved when there is a balance between pro- and anti-inflammatory cytokines [28].

Another family of proteins that acts in the immune system of the cochlea is chemokines. They attract immune cells to protect the tissues of the cochlea during homeostasis. By binding to G-protein-coupled receptors of targeted cells, they trigger signaling pathways between cells to activate them and initialize their movement to a damaged tissue. The chemokine family is also represented by fractalkine, expressed by neurons of spiral ganglia and inner hair cells; monocyte chemoattractant protein 1 (MCP-1), recruiting monocytes and macrophages; chemokine CXCL10, known to be especially active in noise-induced cochlear damage; and CXCL5, with its changing levels in Meniere’s disease and vestibular migraine [29].

Apart from proteins, lipid mediators are also seen in cochlear sites of inflammation. The group is basically represented by prostaglandins (PGD2 and PGE2) and leukotrienes. Prostaglandins show pro-inflammatory action as vasodilators ensuring better blood flow in the cochlea. In contrast, leukotriene LTC4 decreases the blood flow.

The mechanism of the immune system of the inner ear is regulated through the blood–labyrinth barrier. The barrier separates inner ultra-structures of the labyrinth from the systemic circulation and provides a gateway between those two environments. Composed of a basement membrane and endothelial cells connected by pericytes, the blood–labyrinth barrier preserves a balance between the fluids of the inner ear, i.e., potassium-rich endolymph and sodium-rich perilymph. It is also responsible for protecting pathogens and toxins from invading the inside of cochlear ultra-structures, as well as for the transport of cell waste products out. Blood–labyrinth barrier disruption caused by various factors (noise, older age, pathogens, or ototoxic drugs) causes inflammation and activates the immune response [30,31,32]. Determining the mechanisms leading to AIED has been a challenge for many years; however, the progress in immunology has enabled us to better understand the pathology.

### 4.2. Pathology

Autoimmune inner ear disorder is usually associated with progressive sensorineural hearing loss. In some cases, it may be accompanied by vestibular symptoms such as vertigo, dizziness, or tinnitus. It is assumed that the pathology involves an immune response to foreign antigens, which can also be directed against some self-antigens due to molecular mimicry, which is the protein structural similarity between external antigens and the cochlea’s tissue structures. Activation of the immune cascade initiates the inflammation and production of antibodies.

The antibodies’ main targets include cochlin-tomoprotein (CTP), a protein present in the cochlea. The immune response against cochlin is mediated by T cells, and the antigen–antibody complexes cause damage to certain areas of the cochlea, leading to its dysfunction and hearing loss. Another protein present in the cochlea is heat shock protein 70 (HSP 70). This 68-kilodalton protein is an antigen for the anti-HSP 70 antibodies. High levels of anti-HSP 70 antibodies are often detected in AIED patients. Research shows that anti-HSP 70 proteins present a high sensitivity (79%) and high specificity (100%) in AIED cases [33,34].

Cochlear collagen, mainly type II and type IX, has also been identified as a potential self-antigen. As the protein is an important element of the cochlear anatomical structures, an immune response may lead to multifocal inflammation in the inner ear and to serious damage via gathering immune complexes in the small vessels. After the initial activation of the immune reaction, usually in the stria vascularis, the endolymphatic sac is a common site of its further development [35,36,37].

The diagnosis of AIED can be challenging due to a lack of standardized diagnostic criteria and pathognomonic tests. In most patients, some markers are present that may be diagnostic; however, there is no specific testing criteria recommended. The erythrocyte sedimentation rate (ESR) is usually elevated. Anti-heat shock protein 70 (anti-HSP 70) antibodies, antinuclear antibodies (ANAs), and anti-cochlin antibodies are often present. In some AIED cases, lupus anticoagulant antibodies (LA), anticardiolipin antibodies (aCL), and anti-beta2 glycoprotein (anti-B2GP) are detected. Although the above markers are often present in AIED patients, their occurrence is not consistent, and the amounts differ depending on the case. Thus, while they may direct clinical thinking towards AIED, they cannot determine the final diagnosis. Therefore, apart from the lab tests, specific case study, clinical anamnesis, in-depth analysis of clinical data, physical examination, radiological testing, etc., are all necessary for the diagnosis [38,39,40,41].

### 4.3. Treatment

As the pathology of AIED includes inflammation cascade as a background, corticosteroids are commonly used to reduce inflammation. Diaz-Menindez et al. [42] described a group of patients diagnosed with AIED. Of all subjects, 29 patients were subsequently referred to the rheumatology department for steroid treatment, and corticosteroid treatment improved hearing and vestibular symptoms. Methotrexate was the second most used therapy, with 11 of 17 patients reporting a significant improvement. The study showed corticosteroids to be the most effective initial treatment for AIED, which should be followed by other agents to prevent further damage to the cochlea.

As described above, corticosteroids appear to be a first-line treatment in cases of autoimmune inner ear disease. As hearing impairment in AIED may occur solely or in the course of various multisymptomatic and systemic autoimmune conditions, different drug combinations are used depending on the symptom’s spectrum. Therefore, each case should be treated individually, and personalized treatment should be implemented.

Methotrexate was found to be effective in lowering the immune response and improving hearing. Usually used following corticosteroid treatment, it reinforces anti-inflammatory activity, serving as a protective agent against further damage to the cochlea. A methotrexate and azathioprine combination has been successfully used in cases of AIED, as reported by Huang et al. [43].

Different treatment strategies have been used depending on the prevailing manifestations in Susac syndrome. In predominant central nervous symptoms, a combination of prednisone, methotrexate, and sulfasalazine was used. The patients with predominant hearing impairment were treated with oral methylprednisolone and mycophenolate mofetil. In the case of predominant ophthalmological symptoms, treatment included cyclophosphamide and intravenous immunoglobulin administration, as described by Grygiel-Górniak et al. [44].

Leflunomide, combined with steroids, was found to be effective in sudden hearing loss associated with sarcoidosis. This drug combination caused the patient’s hearing to improve significantly, whilst tinnitus was resolved completely, as reported by Chen et al. [45].

In the study of Yellin et al. [46], betahistine benefitted AIED treatment when added to the main therapy. The elimination of hearing loss and vestibular symptoms was faster and more efficient in patients treated with betahistine than in those treated without it.

A certain percentage of AIED cases seem to be resistant to corticosteroids. As described by Vambutas et al. [47], experimental treatment with anakinra appeared to be efficacious in some patients with steroid-resistant AIED. However, continued tests with the drug are needed to gather more information that may be of high clinical importance for the future. Konitsioti et al. [48] published positive results of natalizumab treatment in two patients with Susac syndrome, achieving a significant improvement in hearing.

Based on the literature analysis, although steroids constitute a first choice for treatment in AIED, there are different treatment schemes, which depend mainly on the symptom spectrum. Some schemes are commonly used, whilst others remain under testing.

## 5. Conclusions

The review of the literature shows that AIED is multidisciplinary, related not only to otolaryngology and audiology but also to other medical specialties. Patients with sensorineural hearing loss may be referred to various units for diagnosis and treatment; however, they should be examined holistically, and the diagnostic pathway should include various activities.

The pathology of autoimmune inner ear disease, although better understood recently, is still a subject for further research. Recent studies and the apparent efficacy of corticosteroids and other immunosuppressants in AIED treatment strongly confirm its autoimmune inflammatory origin. Its autoimmune background is also supported by the presence of some biomarkers, which are suggestive of, but not decisive for, the final diagnosis of AIED. However, together with other data, they may direct clinicians to a final diagnosis. In a patient with fast-progressing sensorineural hearing loss in one or both ears, an autoimmune condition should always be considered; therefore, the diagnostic process should be focused on all symptoms reported. Although our knowledge of AIED pathology has improved with the progress in immunology, no biomarker presents sufficient diagnostic accuracy for routine clinical diagnosis of the disease.

As various studies show, treatment strategies vary depending on the symptoms of specific cases. Nevertheless, corticosteroids remain a first-line treatment. Other treatment schemes are used, and some are still experimental, with promising initial results.

## Figures and Tables

**Table 1:** Autoimmune conditions associated with hearing loss and other symptoms.

Disease	Possible Symptoms
**Necrotizing Vasculitis with Polyangiitis** **(Wegener disease)**	Runny nose, nose bleeding, ear infections, crusting in the nose, fever, fatigues, night sweats, glomerulonephritis, otitis media, sensorineural hearing loss, and vertigo
**Linear Morphea**	Skin macules and plaques, pigmentary changes, muscle atrophy, and hearing impairment
**Autoimmune Hepatitis**	Belly discomfort, jaundice, fatigue, sensorineural hearing impairment, liver enlargement, spider angiomas on the skin, joint pain, and skin rash
**Sjogren’s Syndrome**	Dry eyes, dry mouth, fatigue, muscle pain, hearing impairment, and dry skin
**Celiac Disease**	Diarrhea, weight loss, abdominal pain, headache, muscle and joint pain, brain fog, hearing impairment, anemia, and osteoporosis
**Systemic Lupus Erythematosus**	Butterfly rash on the face, migrating joint pain, skin and mucous membranes lesions, sensorineural hearing loss, tinnitus, glomerulonephritis, proteinuria, and erythrocyturia
**Takayasu’s Arteritis**	Weight loss, muscle and joint pain, weak pulse, sensorineural hearing impairment, dizziness, blurry or double vision, and high blood pressure
**Autoimmune Thyroid Diseases**	Chronic fatigue, weight gain, constipation, muscle and joint pain, cold sensitivity, dry skin, hair loss, depression, and problems with memory and concentration
**Susac Syndrome**	Headache, cognitive impairment, memory loss, personality changes, vision impairment, sensorineural hearing impairment, tinnitus, and vertigo
**Polymyositis/Dermatomyositis**	Muscle weakness, fatigue, dysphagia, joint pain and stiffness, skin rash, calcium-filled bumps under the skin, sensorineural hearing loss, dizziness, and vertigo
**Vogt–Koyanagi–Harada Syndrome**	Flu-like symptoms, visual impairment, sensorineural hearing impairment, dizziness, vertigo, nausea, vomiting, alopecia, and vitiligo
**Sarcoidosis**	Chronic cough, chest pain, dyspnea, chronic fatigue, sensorineural hearing loss, pitting oedema, and uveitis
**Rheumatoid Arthritis**	Joint pain and stiffness, joint swelling, sensorineural hearing loss, tinnitus, fatigue, loss of appetite, fever, and cardiac complications
**Multiple Sclerosis**	Fatigue, blurred and double vision, muscle weakness, sensorineural hearing loss, vertigo, electric shock sensation while moving the neck, slurred speech, troubles with memory, and cognitive dysfunction
**Behçet Disease**	Mouth sores, skin lesions and nodules, joint swelling, pain and stiffness, genital sores, headaches and confusion, sensorineural hearing loss, dizziness, and vertigo
**Psoriatic Arthritis**	Redness of the skin, stiffness of the joints, enthesitis, pain in lower back and pelvis, uveitis, sensorineural hearing impairment, and tinnitus
**Cogan Syndrome**	Sensorineural hearing loss, dizziness, vertigo, nausea, vomiting, iritis, eye redness, and lacrimation
**Giant Cell Arteritis**	Headaches, blurred or double vision, jaw claudication, scalp tenderness, fatigue, fever, weight loss, sensorineural hearing loss, vertigo, tinnitus, and dizziness
**Polymyalgia Rheumatica**	Joint pain and stiffness, fatigue, anemia, wrist and knees edema, fever, and flu-like syndromes
**Ulcerative Colitis**	Abdominal pain, cramping, bloody diarrhea, fatigue, fecal urgency, weight loss, sensorineural hearing impairment, and fever
**Ankylosing Spondylitis (Bechterev Disease)**	Joint pain and stiffness of lower back and hips, loss of spinal mobility, sensorineural hearing impairment, uveitis, aortic valve insufficiency, and lung fibrosis
**Serum Disease**	Fever, fatigue, urticaria, joint swelling and pain, face swelling, and sensorineural hearing impairment
**Nodular Arteritis (Polyarteritis Nodosa)**	Skin tenderness, nodules under the skin, fatigue, weight loss, joint pain, and sensorineural hearing impairment

## Data Availability

No new data were created or analyzed in this study. Data sharing is not applicable to this article.

## Abbreviations

The following abbreviations are used in this manuscript:

AIED	Autoimmune inner ear disease
SNHL	Sensorineural hearing loss
IL-6	Interleukin type 6
IL-1β	Interleukin type 1β
Il-17	Interleukin type 17
TNF-α	Tumor necrosis factor α
IL-10	Interleukin type 10
IL-22	Interleukin type 22
IL-33	Interleukin type 33
TGF-β	Transforming growth factor β
MCP-1	Monocyte chemoattractant protein 1
CXCL 10	C-X-C motif chemokine ligand 10
CXCL 5	C-X-C motif chemokine ligand 5
PGD2	Prostaglandin D2
PGE2	Prostaglandin E2
LTC4	Leukotriene C4
CTP	Cochlin-tomoprotein
HSP-70	Heat shock protein 70
ESR	Erythrocyte sedimentation rate
ANA	Antinuclear antibodies
LA	Lupus anticoagulant antibodies
aCL	Anticardiolipin antibodies
anti-B2GP	Anti-beta 2 glycoprotein
CD 163	Protein CD 163
AIH	Autoimmune hepatitis
SLE	Systemic lupus erythematosus
TAK	Takayasu’s arteritis
PM/DM	Polymyositis/dermatomyositis
VKH	Vogt–Koyanagi–Harada syndrome
RA	Rheumatoid arthritis
MS	Multiple sclerosis
AITDs	Autoimmune thyroid diseases
TPO	Thyroid peroxidase
Tg	Thyroglobulin
TRAb	Anti-thyrotropic receptor antibodies
TgAbs	Anti-thyroglobulin antibodies
TPOAbs	Anti-thyroid peroxidase antibodies
TRAbs	Anti-thyrotropic receptor antibodies
BD	Behcet disease
GPA	Necrotizing vasculitis with polyangiitis (Wegener’s disease)
GCA	Giant cell arteritis
PMR	Polymyalgia rheumatica
PA	Psoriatic arthritis
AS	Ankylosing spondylitis (Bechterev disease)
SD	Serum disease
CU	Ulcerative colitis (colitis ulcerosa)
PNA	Polyarteritis nodosa
